# TEM8 in Oncogenesis: Protein Biology, Pre-Clinical Agents, and Clinical Rationale

**DOI:** 10.3390/cells12222623

**Published:** 2023-11-14

**Authors:** Samuel A. Kareff, Virginia Corbett, Paul Hallenbeck, Aman Chauhan

**Affiliations:** 1 University of Miami Sylvester Comprehensive Cancer Center/Jackson Memorial Hospital, Miami, FL 33136, USA; 2 Mount Sinai Tisch Cancer Center, New York, NY 10029, USA; 3 Seneca Therapeutics, Philadelphia, PA 19103, USA; phallenbeck@senecatherapeutics.com; 4 Division of Medical Oncology, Department of Medicine, University of Miami Sylvester Comprehensive Cancer Center, Miami, FL 33136, USA

**Keywords:** TEM8, *ANTXR1*, oncogenesis

## Abstract

The TEM8 protein represents an emerging biomarker in many solid tumor histologies. Given the various roles it plays in oncogenesis, including but not limited to angiogenesis, epithelial-to-mesenchymal transition, and cell migration, TEM8 has recently served and will continue to serve as the target of novel oncologic therapies. We review herein the role of TEM8 in oncogenesis. We review its normal function, highlight the additional roles it plays in the tumor microenvironment, and synthesize pre-clinical and clinical data currently available. We underline the protein’s prognostic and predictive abilities in various solid tumors by (1) highlighting its association with more aggressive disease biology and poor clinical outcomes and (2) assessing its associated clinical trial landscape. Finally, we offer future directions for clinical studies involving TEM8, including incorporating pre-clinical agents into clinical trials and combining previously tested oncologic therapies with currently available treatments, such as immunotherapy.

## 1. Introduction

The TEM8 protein represents an emerging biomarker for the diagnosis and prognosis of many solid tumor histologies. Initially discovered in 2000 as a component of colorectal tumor vascular endothelium [1], TEM8 has since been characterized and implicated in several biological pathways. For example, the protein binds the protective antigen subunit of the toxin complex of *Bacillus anthracis*, thereby eliciting pathogenicity via the lethal factor (LF) and edema factor (EF) toxins [2]. Moreover, it is a unique receptor in that TEM8 is overexpressed by mammalian neoplastic tissues [3].

In addition to the various roles it plays in oncogenesis, including angiogenesis, epithelial-to-mesenchymal transition, and cell migration, it has also been shown that the overexpression of the *ANTXR1* gene that codes for the protein TEM8 correlates with poor prognostic indicators in several solid tumor histologies. As such, TEM8 has been identified as the target of novel oncologic therapies. It is especially attractive given its selective expression on the surface of solid tumor cells and associated stromal cells, such as cancer stem cells, invasive cancer cells, and immune cells, such as macrophages, angiogenic endothelial cells, pericytes, and cancer-associated fibroblasts [4].

We review herein the role of TEM8 in oncogenesis. First, the physiologic structure and function of TEM8 is reviewed. Next, the diverse roles that the receptor plays in both physiologic pathways and tissues, including as a key regulator of collagen synthesis via the Wnt/β-catenin signaling pathway, as a key player in metabolomics, and as a mechanosensor in the bone marrow, are discussed. We then summarize the known pathophysiologic functions that TEM8 serves in cancer and related cell types. The pre-clinical and clinical data currently available are then synthesized by highlighting the protein’s prognostic and predictive abilities in various solid tumors and assessing the current clinical trial landscape that features agents involving TEM8. Finally, we offer future directions for clinical studies involving TEM8.

## 2. TEM8 Structure and (Patho)physiologic Functions

### 2.1. The Physiologic Roles of TEM8

TEM8 is a unique protein with several (patho)physiologic functions in cancer. It is upregulated in the setting of environmental stress in several tissue types and, thereby, superficially decreases growth factors to play a role in cell adhesion, angiogenesis, collagen metabolism, and the metastatic spread of cancer [5]. The protein’s complex integrin-like structure [6] and its many functions [7] have been reported in detail previously; however, we will review some key characteristics.

The TEM8 structure includes an extracellular domain containing a conserved magnesium-coordinated, metal ion-dependent adhesion site (MIDAS) that permits an integrin-like open conformation [8]. Once activated, the TEM8 extracellular domain impairs the growth of various human tumor xenografts, thereby inhibiting angiogenesis [9]. TEM8 is expressed on the endothelial and other stromal cells of various tumors [10]. However, human tissues may employ different variants that explain the different oncogenic roles that TEM8 plays in various tumor histologies (see Section 2.3 below).

### 2.2. The Role of TEM8 in Key Pathways: Wnt/β-Catenin, Metabolomics, and Mechanosensing in the Bone Marrow

TEM8 functions in several key physiologic pathways, such as the Wnt/β-catenin cascade and glutamine metabolism. The Wnt/β-catenin signaling cascade controls cell proliferation, polarity, and migration [11]. TEM8-expressing cells induce this pathway substantially via endothelial cell–matrix interactions with collagen, thereby increasing human umbilical vein endothelial cells (HUVEC) initiation for angiogenesis via the induction of a more reactive and mobile state [12].

The greatest amount of metabolomics information known about TEM8 involves glutamine metabolism. TEM8’s role in tumorigenesis involves catalyzing the breakdown of glutamine as a molecular substrate and is reviewed below [5]. TEM8 has also been found to interact with isoenzymes of pyruvate kinase [13], which can promote reductive glutamine metabolism in pyruvate kinase-knockout cells [14].

The bone marrow is a unique tissue in which TEM8 plays a significant role. The osteoblastic-differentiating GATA2 transcription factor binds to the promoter region of TEM8 to regulate its expression preferentially [15]. TEM8 next regulates receptor activator of nuclear factor kappa B ligand (RANKL)-induced osteoclast differentiation, thereby modifying bone resorption [16]. Interestingly, the genetic manipulation of TEM8 within bone marrow-specific macrophages modulates capillary-like tube formation in HUVEC through a section of angiogenic molecules, including VEGF-A [16]. Finally, TEM8 may act independently as a novel mechanosensor that shepherds the differentiation of bone marrow stromal cells into chondroblasts via changes in hydrostatic pressure [17]. This exclusive signaling pathway may act independently from TEM8’s role as an integrin-like protein [17].

### 2.3. The Pathiophysioloic Roles of TEM8 in Cancer

Once expressed on the epithelia of tumor cells, TEM8 plays several pathophysiologic roles. First, tumors utilize TEM8 in similar pathophysiologic manners, given its physiologic participation in the Wnt/β-catenin signaling cascade. Accordingly, the knockdown of this receptor decreased cell proliferation, migration, and invasion and induced apoptosis in lung adenocarcinoma [18] and hepatocellular carcinoma models [19]. There are additional mechanisms by which TEM8 is thought to exert these effects. In glioblastoma models, TEM8 induced chemo-radioresistance by stabilizing β-catenin via the activation of a transcriptional program [20]. Protective antigens applied during elevated moments of TEM8 expression reduce vascular density via Wnt antagonism [21], implying that TEM8 alone can regulate vessel proliferation. Finally, natural ligand C5a may activate TEM8 via Wnt signaling to elevate Wnt target genes in mammosphere assays [22].

TEM8 has a unique relationship with tumor-associated macrophages (TAM) in the tumor microenvironment (TME). *ANTXR1* expression is known to correlate with cancer-associated fibroblasts and TAM, which allows for phenotypic differentiation into M1 or M2 macrophages, as well as the secretion of immunosuppressive factors [23]. As discussed below, TAM are also a confirmed portal of entry into tumoral tissues.

Increasing evidence shows that TEM8’s function is crucial for various oncologic processes, including collagen synthesis, as mentioned above. In addition to its role as a complex integrin-like molecule, TEM8 may regulate collagen synthesis during tumorigenesis. This relationship has been reviewed previously [7]. The proposed relationship relies on the ability of the tumor-associated stroma to degrade collagen into amino acids, such as glutamine, thereby facilitating the cancer cells’ use of glutamine under starvation conditions [5]. TEM8 is the main catalyst of collagen breakdown in this mechanism.

**Figure 1:**
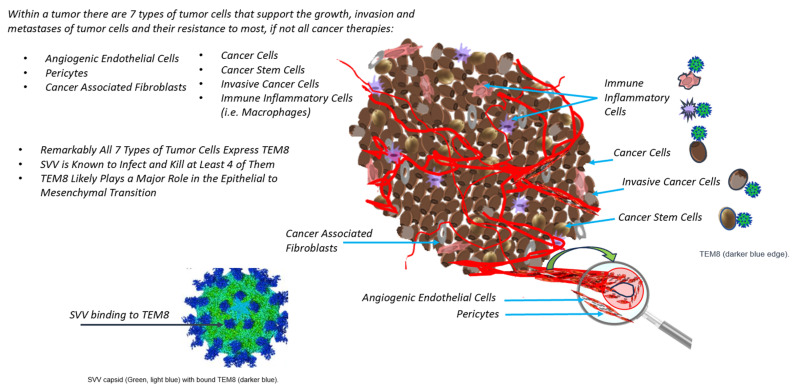
This figure demonstrates the various types of tumor cells, including angiogenic endothelial cells, pericytes, cancer-associated fibroblasts, cancer cells, cancer stem cells, invasive cancer cells, and immune inflammatory cells, in which the TEM8 receptor plays a pathophysiologic role.

TEM8 may interact with collagen in additional pathogenic pathways. For example, the α3 subunit of collagen VI interacts with TEM8 [10], which later promotes the migration of endothelial cells [24]. Accordingly, neoplastic cells exploit this process via TEM8 cell surface proteins in the tumor-associated stroma, as described above [5]. Cancer stem-like cells utilize TEM8 interactions with its ligand C5a to bridge collagen cleaving in order to remodel the TME for metastatic progression [22]. *RUNX2*, another oncogenic driver, can also regulate TEM8 to increase its expression in cartilaginous tissues, thereby causing chondrocyte apoptosis and matrix mineralization [25]. Progressive fibrosis in the skin and other organs can be seen when TEM8 loses function within fiber-forming collagens, leading to subsequent hyperproliferative and leaky blood cells in tissues such as the skin [26]. Additional pathways, such as that of connective tissue growth factor (CTCF), may contribute to VEGF-dependent increases in collagen type 1 expression [5]. The interplay between collagen and cancer cell metabolism is becoming more understood, and TEM8 modulation may serve as a key driver in this synergy [27]. See Figure 1 for a summary of the known functions that TEM8 serves in cancerous tissues.

## 3. Review of Pre-Clinical and Clinical Data Surrounding TEM8

Both pre-clinical and clinical data support the known evidence regarding TEM8’s role in tumorigenesis. Importantly, TEM8 has emerged as a prognostic and possible predictive biomarker in several solid cancers. We review the available pre-clinical and clinical data below.

### 3.1. Pre-Clinical Data

Given the plethora of physiologic pathways and pathophysiologic functions that TEM8 exploits, pre-clinical studies have attempted to identify novel targets and treatments. Various therapeutic strategies have been employed to intercept TEM8, including direct pathway inhibitors, RNA targeting, DNA-based vaccines, oncolytic viruses, antibody and antibody-like molecules, antibody–drug conjugates, and CAR-T cells.

Direct pathway inhibitors seek to overcome upstream or downstream targets from TEM8-binding cells. Inhibitors targeting RAC1 (EHop-016) and MEK (PD98059) have shown efficacy in suppressing malignant tendencies in ovarian cancer cells that overexpress TEM8 in vitro via the Rac1/Cdc42/JNK and MEK/ERK/STAT3 pathways [15]. Similarly, an H3K27me2 inhibitor (GSK126) inhibits the effects of deoxynivalenol, a mycotoxin, on the expression of TEM8, thereby decreasing cell migration [28].

DNA and RNA targeting, as well as DNA vaccines, have also demonstrated efficacy. Targeted homologous recombination of the *ANTXR1* gene resulted in impaired tumor growth of transplanted melanoma and other tumors in mouse models [29]. Interestingly, the CRISPR/Cas9-mediated ablation of TEM8 in tumor cells had little impact on tumor growth, though this observation is only yet observed during in vitro testing [30]. An interference vector encoding short hairpin RNA (shRNA) targeting TEM8 inhibited cell proliferation, thereby increasing cell apoptosis by arresting proliferating cells in the G1 phase to block cell migration [31]. Similarly, a lentiviral vector that encodes shRNA inhibited tumor growth and displayed anti-angiogenic properties in a lung cancer murine model [32]. Finally, a DNA vaccine encoding TEM8 and its extracellular domain was tested in combination with rat neu and human tyrosinase-related protein 1 (hgp75) DNA vaccines [33]. This treatment combination relied on synergy provided by CD8+ T cells in murine breast cancer and melanoma models, resulting in anti-tumor effects such as increased inflammation and tumor necrosis [33].

Oncolytic viruses continue gaining attention as they demonstrate pre-clinical and clinical efficacy in various tumor types. A recombinant adenovirus (Ad-TEM8) transduced dendritic cells that successfully secreted interferon-γ to lyse hepatocellular carcinoma cells in murine models [34]. Ad-TEM8 induced antitumor immunity by disrupting the tumor vasculature, thereby increasing mice’s life span [34]. A novel oncolytic virus, Senecavirus A (SVV-A) (Seneca Therapeutics; Blue Bell, PA, USA), is further discussed below, as it has already entered clinical trials. However, there are compelling data that it can overcome immune checkpoint inhibitor (ICI) resistance (i.e., PD-1/PD-L1 and CTLA4 antibodies) in pancreatic cancer murine models, thereby converting immunologically “cold” tumors via systemic anti-tumor immune responses with increased T-cell infiltration [35,36]. It had previously been shown to colonize porcine macrophage PAM-Tang [37] and 3D4/21 [38] cell lines.

Antibodies, antibody-like molecules, and antibody–drug conjugates have also demonstrated success. Two anti-TEM8 monoclonal antibodies that require conjugation to a saporin toxin, AF334 and SB5, recognize varying forms of TEM8 on cell surfaces in order to eliminate TEM8-positive cells [39]. Another antibody-like molecule encoding the protective antigen-binding domain of TEM8 that links to its Fc IgG1 was able to protect macrophage-like cells in a dose-dependent manner, thereby suppressing growth and metastases in xenograft human tumors in mouse models [13]. A tri-specific killer engager targeting TEM8 via natural killer cell induction and IL-15 co-stimulation led to anti-stroma and anti-angiogenic effects in tumor cells and stroma [40]. A novel antibody–drug conjugate targeting TEM8 elicited drug activation and release through a stroma mechanism within the TME [30]. This agent demonstrated regression and eradication of multiple solid tumor types, prolonging overall survival in xenograft models [30]. Finally, L2, a therapeutic anti-TEM8 monoclonal antibody, can be used as a medium in PET imaging studies when coupled with radioactive zirconium (^89^Zr) in mouse tumor models of colorectal adenocarcinoma, gastric carcinoma, breast ductal carcinoma, epidermoid carcinoma, and glioblastoma [41].

The role of CAR-T cells continues evolving. One pre-clinical model of CAR-T cells based on TEM8-secreting immunostimulatory cytokines demonstrated success in targeting both tumor endothelial cells and TEM8-expressing triple-negative breast cancer cells by blocking tumor neovascularization [42]. Another model confirmed in vitro responses in mouse models [43].

### 3.2. Clinical Data

TEM8 is already known to be upregulated in several tumor models, including but not limited to melanoma (mice [24,33]), triple-negative breast cancer (mice [19]; human [42]), undifferentiated breast cancer (human [44]; mice [33]), medullary and estrogen receptor-negative breast carcinomas (human [22]), prostate (human [45]), pancreatic ductal adenocarcinoma (xenograft [46]), gastric (human [23]), and colorectal cancers (human [47]).

Accordingly, retrospective clinical studies have sought to estimate any prognostic or predictive abilities of the TEM8 biomarker. Regarding clinicopathologic characteristics, TEM8 is associated with worse clinical outcomes. These include but are not limited to nodal involvement or disease progression in breast cancer [44]; tumor size and AJCC staging in lung adenocarcinoma [18]; therapeutic resistance in prostate cancer [45]; increased mortality in pancreatic ductal adenocarcinoma [46]; TNM staging and the presence of lymphovascular invasion, the depth of invasion, and the presence of lymph node or distant metastases in colorectal cancer [47]; and adverse clinicopathologic characteristics, resistance to chemotherapy, and lower tumor mutational burden in gastric cancer [23].

Importantly, poor clinical outcomes have been quantified based on TEM8 expression levels. These findings are listed in Table 1. In summary, increased TEM8 levels have been shown to lead to decreased overall survival in lung cancer [18,24], angiosarcoma [48], colorectal cancer [47], gastric cancer [23], prostate cancer [45], and ovarian cancer [15]. One exception to this trend is TEM8 expression in hepatitis B (HBV)-induced hepatocellular carcinoma in which *ANTXR1* is hypothesized to act as a cellular receptor for HBV and transport unknown antineoplastic therapeutics within the cell [49]. Regardless, it is clear that TEM8 expression is generally associated with more aggressive disease biology, such as tumor invasion and metastases, and poor clinical outcomes, including resistance to chemotherapy, radiation therapy, and ICIs.

The clinical trial landscape involving TEM8 continues evolving and is detailed below in Table 2. After initial interest in targeting the TEM family was reported in 2004 [10], at least four known clinical trials have either been completed or are in the process of evaluating agents involving TEM8. Three trials involve a novel oncolytic virus named Seneca Valley Virus (SVV-001, NTX-010, or SVV-A) (Seneca Therapeutics; Blue Bell, PA, USA). This non-enveloped RNA virus—the only member of the Senecavirus A (SVA) species in the Senecavirus genus and Picornaviridae family—has been reviewed in detail previously in the context of its receptor TEM8 [3,4]. In brief, the oncolytic virus exploits the TEM8 receptor to invade human tumor cells via N-linked glycosylation in the TEM8 vWA domain [6,50]. Additional co-factors help facilitate virus attachment and cell entry, such as metal ions in the *ANTXR1* domain, as well as serine-to-alanine mutations [51]. After cell entry, robust SVV-001 replication requires additional innate immune mechanisms, including decreased expression of antiviral interferon genes to permit viral entry [3].

The Phase I SVV-001 study confirmed safety in cancer patients; however, the Phase II study in SCLC failed to meet its primary endpoint. This is likely attributed to a single intravenously administered dose of SVV-001, resulting in neutralizing antibodies and patient selection of a biomarker-unselected population. Neutralizing antibodies are innate defense mechanisms generated to eliminate future encounters with pathogens, such as viruses [55], and are reported mechanisms to resistance of investigational therapeutics such as monoclonal antibodies. Pre-clinical data suggest that multiple intra-tumoral administrations may overcome this resistance pathway [35]. Moreover, biological phenomena such as intra-tumoral heterogeneity may diminish the efficacy of targeted agents in otherwise biomarker-unselected populations [56].

Despite these drawbacks, promising signals of efficacy were noted. In the first Phase 1 study reported by Rudin et al., SVV-001 was well tolerated in solid tumors with neuroendocrine features without dose-limiting toxicities [54]. The authors noted that neutralizing antibodies directed against SVV-001 developed in all patients, though it is unclear if such antibodies would prohibit activity after a single systemic or intra-tumoral dosage, given its intravenous administration. Moreover, there was a long-term response (progression-free survival [PFS] of 10 months and overall survival [OS] of greater than 3 years) in a patient with chemo-refractory small cell lung cancer (SCLC) [54]. Another Phase 1 study in various solid cancers, including neuroendocrine tumors, investigated dose-limiting toxicity in the pediatric population in conjunction with cyclophosphamide. The study observed about a 67% stable disease rate [53]. Most patients also developed neutralizing antibodies within three weeks of intravenous administration that did not appear to be eradicated by cyclophosphamide. Given the agent’s signal in SCLC, a Phase 2 trial investigating NTX-010 in patients who received platinum doublet chemotherapy and achieved stable disease or progression of disease did not find a difference in PFS compared to placebo [52]. Interestingly, detectable viremia for up to 2 weeks after treatment was statistically associated with shorter PFS and OS. This is probably due to the selection of a TEM8-positive patient population, which is now understood to confer an extremely poor prognosis. This finding suggests that viral titer monitoring may serve as a biomarker for disease progression in this otherwise therapy-resistant therapeutic setting with poor outcomes [52].

## 4. Future Directions for Research Involving TEM8

The oncogenic roles of TEM8 are diverse and might be targeted by oncolytic therapies in many ways. However, additional research is needed to ensure that pre-clinical findings and clinical signals translate into meaningful therapeutic progress. First, researchers might validate this biomarker in other solid tumors in future clinical trials. Indeed, a Phase 2 study (NCT019733322) involving vaccination with autologous dendritic cells combined with immunomodulated radiotherapy and/or preleukapheresis interferon-alfa in metastatic melanoma is being conducted to explore TEM8/*ANTXR1* upregulation. Additionally, the authors of this review are planning retrospective analyses to review the association between the expression of the TEM8 biomarker and clinical outcomes in multiple tumor types, especially neuroendocrine histologies.

Regarding future clinical trials, researchers might consider using either novel TEM8-targeting agents or innovative combination therapies involving recently adopted treatment paradigms. This review has demonstrated multiple pathophysiologic roles that TEM8 plays in tumorigenesis and has focused on collagen synthesis, the associated Wnt/β-catenin pathway, and metabolomics. These tumor-associated or TME actors represent potential avenues for clinical research, given the receptor’s presence on the surface of solid tumor cells as well as its partners, such as cancer stem cells, invasive cancer cells, immune cells such as macrophages, angiogenic endothelial cells, pericytes, and cancer-associated fibroblasts (see Figure 1). For example, TEM8-expressing cancer-associated fibroblasts have recently been shown to confer resistance to immunotherapy in breast cancer models [57]. Additionally, there are several pre-clinical drugs and other targets that have yet to be evaluated in clinical studies. As detailed above, these include direct pathway inhibitors, RNA targeting, DNA-based vaccines, antibody and antibody-like molecules, antibody–drug conjugates, and CAR-T cells. Sadly, none of these therapeutic agents have yet advanced to clinical trials. However, given the recent success of TEM8-targeting agents in murine models, such as the intra-tumoral injection of SVV into treatment-refractory pancreatic cancers and the subsequent rendering of an immunotherapy response due to enhanced T-cell infiltration [35], additional investigation is warranted. Finally, the prospective evaluation of biomarker-selected populations based on TEM8 expression may reveal which patients might benefit most from TEM8-targeting agents.

Oncolytic viruses demonstrate the most solid clinical evidence to date; however, they have not yet met primary endpoints in previous clinical trials. They, too, ought to be tested in novel combinations to improve outcomes, such as in conjunction with ICIs [58]. One might imagine leveraging this treatment strategy to combine immunotherapy, such as PD-(L)1 and CTLA-4 inhibitors, with oncolytic viruses. Indeed, a Phase 1 trial combining the intra-tumoral administration of SVV with ICIs targeting PD-(L)1 and CTLA4 is anticipated to initiate the enrollment of treatment-refractory neuroendocrine neoplasms within months after the publication of this review. Moreover, this trial highlights novel therapeutic exploitation of the TME by recruiting additional immune system actors (e.g., inflammatory T-cells) to boost the efficacy of immunotherapy [36].

No matter which strategy is employed, it is clear that there remains much to learn about exploiting the TEM8 receptor in treating human cancers. This treatment paradigm becomes especially attractive when considering that the receptor is linked to seven cancer cell types, for example, cancer-associated fibroblast CAF-S1 clusters, which have been associated with resistance to immunotherapy in in vitro melanoma and NSCLC samples [57]. In brief, the TEM8 receptor will likely serve as a novel therapeutic marker in many trials to come, given its multiple pathophysiologic functions and unique expression in cancerous cells.

## Figures and Tables

**Table 1:** List of studies evaluating prognostic or predictive measures of TEM8/*ANTXR1* expression.

Study Lead Author (Year)	Study Title	Study Conclusion
Sun et al., (2021) [24]	The relationship between TEM8/*ANTXR1* and early diagnosis and prognosis of lung cancer	High vs. Low TEM8/*ANTXR1* expression led to 58.29% vs. 68.69% 3-year overall survival, with a worse prognosis overall.
Kusaba et al., (2021) [48]	Overexpression of tumor endothelial marker 8 protein predicts poor prognosis in angiosarcoma	High vs. Low TEM8 expression led to 14 vs. 22 days median overall survival.
Pietrzyk et al., (2021) [47]	Clinical Value of Detecting Tumor Endothelial Marker 8 (*ANTXR1*) as a Biomarker in the Diagnosis and Prognosis of Colorectal Cancer	High vs. Low TEM8/*ANTXR1* expression led to a median overall survival of 27 months vs. estimate not reached.
Si et al., (2021) [49]	*ANTXR1* as a potential prognostic biomarker for hepatitis B virus-related hepatocellular carcinoma identified by a weighted gene correlation network analysis	High vs. Low *ANTXR1* expression led to a median overall survival of 2540 days vs. 555 days.
Huang et al., (2020) [23]	*ANTXR1* is a Prognostic Biomarker and Correlates with Stromal and Immune Cell Infiltration in Gastric Cancer	Cohort 1 (TCGA): High vs. Low *ANTXR1* expression resulted in 25 vs. 55 months overall survival.Cohort 2 (ACRG): High vs. Low *ANTXR1* expression led to 31 months vs. estimate not reached.
Ding et al., (2021) [18]	Tumor Endothelial Marker 8 Promotes Proliferation and Metastasis via the Wnt/β-Catenin Signaling Pathway in Lung Adenocarcinoma	High vs. Low TEM8 expression led to 33 vs. 49 months of overall survival.
Li et al., (2021) [45]	*N-Myc* promotes angiogenesis and therapeutic resistance of prostate cancer by TEM8	High vs. Low TEM8 expression led to 115 vs. 174 months overall survival.
Wang et al., (2020) [15]	Overexpression of TEM8 promotes ovarian cancer progression via Rac1/Cdc42/JNK and MEK/ERK/STAT3 signaling pathways	High vs. Low TEM8 expression led to 71 months vs. estimate not reached.

Table alt text: This table shows the study lead author (with the corresponding year of publication), title, and prognostic or predictive endpoints for all currently available clinical data that evaluate survival in the context of TEM8 and/or *ANTXR1* expression. For studies that did not explicitly list survival endpoints in the article text, estimates for survival were extrapolated using the publicly available Web Plot Digitizer tool, available at https://automeris.io/WebPlotDigitizer (accessed on 25 May 2023).

**Table 2:** List of current and previous clinical trials involving TEM8/*ANTXR1*.

Trial Name (NCT)	TEM8/*ANTXR1* Involvement	Predicted or Reported Outcomes
Vaccination with Autologous Dendritic Cells Loaded with Autologous Tumor Lysate or Homogenate Combined with Immunomodulated Radiotherapy and/or Preleukapheresis IFN-alfa in Patients with Metastatic Melanoma: a Randomized “Proof-of-principle” Phase II Study (NCT019733322)	Measuring levels of TEM8/*ANTXR1* upregulation	The investigators plan to report the biological effects of preleukapheresis IFN-alfa on TEM8/*ANTXR1* upregulation at the mRNA level upon dendritic cells’ maturation by flow cytometry and real-time PCR.
Seneca Valley Virus-001 After Chemotherapy in Treating Patients with Extensive-Stage Small Cell Lung Cancer (NCT01017601)	Virus (NTX-010) uses TEM8 to enter the cell.	Schenk et al. (2020) [52] reported no change in the primary endpoint, PFS, for all patients treated after platinum doublet chemotherapy.
Seneca Valley Virus-001 and Cyclophosphamide in Treating Young Patients with Relapsed or Refractory Neuroblastoma, Rhabdomyosarcoma, or Rare Tumors with Neuroendocrine Features (NCT01048892)	Virus (NTX-010) uses TEM8 to enter the cell.	Burke et al. (2014) [53] reported that NTX-010 was feasible and tolerable at three dose levels alone and in combination with cyclophosphamide. However, neutralizing antibodies developed in the majority of patients.
Safety Study of Seneca Valley Virus in Patients with Solid Tumors with Neuroendocrine Features (NCT00314925)	Virus (SVV-001) uses TEM8 to enter the cell.	Rudin et al. (2011) [54] reported no dose-limiting toxicities for the desired dose as well as response in small-cell lung cancer. Neutralizing antibodies developed in all patients.

Table alt text: This table lists the trial name (and NCT registration number), corresponding TEM8/ANTXR1 involvement, and predicted or reported outcomes for previous and current trials concerning TEM8/ANTXR1 registered on https://clinicaltrials.gov (accessed on 25 May 2023).
